# Aneurism of the Aorta

**Published:** 1899-11

**Authors:** W. M. L. Coplin, E. A. Thornton


### Selection.

---

### ANEURISM OF THE AORTA.

BY W. M. L. COPLIN, M. D., AND E. A. THORNTON, M. D.

[*From Proceedings of the Pathological Society of Philadelphia.*]

LARGE saccular aneurism of the ascending and beginning of the transverse part of the arch of the aorta, showing old rupture into the pulmonary artery, and more recent, fatal rupture into the pericardium; pleurisy, atelectasis, pericarditis.

Clinical history by Dr. Thornton: T. B., male, aged 50 years; occupation, painter. The subject of these notes was addicted to the use of alcohol. He had no symptoms of chronic lead-poisoning, and no lead was ever found in the urine. In September, 1897, he began to experience pain in the precordial region and underneath the sternal notch; the pain also radiated down the left arm. In October of that year he exhibited a pulsating tumor, with all the usual accompanying signs and symptoms of aneurism of the arch of the aorta. He refused to discontinue his occupation, and continued to do the more or less laborious work of a painter, with an occasional lapse of a few days into his old habit of drink. The symptoms all became more pronounced. Dyspnea was at all times very pronounced upon the least exertion, and he had some difficulty in swallowing solid food.

In February, 1898, he consented to give up all work, and was put to bed. Treatment was continued for three months, and at the expiration of this time he was much more comfortable, and the pulsation could no longer be felt, nor could any bruit be heard. One symptom had become more marked—namely, greater difficulty in swallowing solid food. Stress of circumstances compelled him to resume his usual work, which he continued to pursue until within about four weeks of his death, which occurred upon April 2, 1899. On March 1st, during a severe coughing spell, he became faint and was overcome by an attack of syncope, from which he slowly recovered. On March 14th he was taken with an attack of pleurisy with effusion. On March 28th the left chest was aspirated and 1500 c.c. of clear, serous fluid (not blood-stained) removed. During the night of April 1st he was taken with syncope, precordial pain, and greatly embarrassed respiration, quickly followed by death.

Abstract of post mortem notes by Dr. Coplin: Post-mortem held thirty-eight hours after death. Body of man past middle age, fairly well nourished. There was a slight amount of pretibial edema. The subcutaneous fat of the trunk and abdomen was slightly edematous; the musculature of the chest and abdomen on section seemed normal, but slightly pale. The intercostal spaces were prominent on the left side, less so on the right. The peritoneal cavity contained a small quantity of clear, straw-colored fluid.

The left pleura contained 400 c.c. of slightly blood-stained, cloudy fluid. The serous surface was covered by a thin layer of fibrin which was, at points, slightly villous. The right pleural cavity contained 600 c.c. of clear fluid, which was very faintly blood-tinged.

The serous membrane was smooth; there were a number of old adhesions at the apex.

The pericardial cavity contained 700 c.c. of fluid, which was intensely red and contained a number of soft, red, post-mortem clots. The pericardium shows a marked villous, fibrinous deposit, abundantly present over both parietal and visceral layers. At the upper and outer aspect of the pericardial cavity there is an opening fully occupied and apparently closed by a post mortem clot. This opening is slit-like, 2 cm. in length, with ragged edges, and lies immediately adjacent to and parallel with the pulmonary artery. It communicates with a globular tumor continuous with the aorta. This tumor, evidently an aneurism, occupies the superior and middle mediastinum, pressing backward upon the esophagus and bronchi and anteriorly upon the sternum, which was slightly eroded. The sac is apparently globular, its external diameter is 20.5 cm. It is firm over the whole surface. It lies in immediate relation with the upper lobes of both lungs the bronchi and great veins, the esophagus, and two vertebræ, which were slightly eroded, and the sternum.

The thoracic visceræ, with the exception of the esophagus were removed *en masse*. The bronchi were not perceptibly collapsed.

There is considerable subpericardial fat, and this, at points, appears to extend into the muscle. The myocardium seems slightly softer than normal, and on section appears to show slight striation or mottling. The cavities of the heart are moderately distended, the right more than the left. The orifices and valves of the right side are normal. The mitral valves show slight thickening with atheroma at the base. The aortic orifice transmits four fingers. The valves show some old fenestræ above the line of contact; they are slightly thickened, and show some atheroma and fibrous thickening extending into the sinuses of Valsalva. The coronary arteries are normal. Beginning 1 cm. from the aortic orifice there is a globular mass completely surrounded by a laminated white thrombus. Anterior to the right side of the cavity the thrombus is much thicker. The cavity occupied by the thrombus measures 16.5 cm. in diameter; its wall aside from the laminated thrombus is about 4 mm. in thickness; the disparity between the external and internal measurements is due to a slight reduction in the size of the mass after opening. Anteriorly and to the right the thrombus is 8 cm. in thickness, while to the left and over the pulmonary blood-vessels it is less than 1 cm. in thickness. Just over the pulmonary artery it is barely 0.25 cm.

in thickness and communicates with the pulmonary artery, 4 cm. from the pulmonary orifice, by an irregular, jagged, button-like projection for about 1 cm. The opening in the pulmonary artery is 1.5 cm. in length, but on attempting to remove the thrombus this opening is extended to a distance of 3 cm.; apparently the wall of the pulmonary artery has been replaced by the thrombus; 1 cm. external to this opening is the opening into the pericardium, already referred to. The canal through the thrombus is a flattened cylinder, ovoid on transverse section, which barely transmits three fingers, measuring 6.5 cm. in width by 1.5 cm. in the opposite direction. The aorta immediately above the valves shows considerable atheroma; where it leaves the aneurism it is narrowed into a circular orifice, which barely transmits the index finger. The orifice is surrounded by a densely thickened band which cuts with very great resistance. The lower part of the thoracic aorta is normal. The heart, pericardium, and fragments of thrombi weigh 1880 grammes.

Both lungs showed areas of atelectasis with considerable hypostatic congestion. The bronchial mucosa was red and edematous, and many of the smaller bronchi were apparently occluded by plugs of mucus. With the exception of a slight degree of red atrophy of the liver and a moderate amount of induration in the kidneys the other organs show nothing worthy of special consideration.

Multiple ruptures of an aneurism are not extremely uncommon. Rupture into the pulmonary artery must be very rare. Rupture into a pulmonary vein is not so infrequent. That the rupture into the pulmonary artery is old, and the rupture into the pericardium recent, is easily demonstrated. Whether the rupture into the pulmonary artery dates back to the period when the patient first went to bed, February, 1898, or whether it occurred at the beginning of his last illness cannot be accurately determined. The rupture is not in a condition which would enable one to say, with any degree of definiteness, as to when it occurred; it must, however, have been a question of some weeks. It is possible that the fainting spell which occurred on March 1st may have been due to the sudden establishing of communication between the aneurism and the pulmonary artery.

---

CURED.—He—I was cured by the faith cure.
She—What was your ailment?
He—Faith in the faith cure.

—*Brooklyn Life.*

